# Few-shot image classification algorithm based on attention mechanism and weight fusion

**DOI:** 10.1186/s44147-023-00186-9

**Published:** 2023-03-02

**Authors:** Xiaoxia Meng, Xiaowei Wang, Shoulin Yin, Hang Li

**Affiliations:** Software College of Shenyang Normal University, Shenyang, China

**Keywords:** Image classification, Few-shot learning, Metric-based method, Attention mechanism, Weight fusion

## Abstract

Aiming at the existing problems of metric-based methods, there are problems such as inadequate feature extraction, inaccurate class feature representation, and single similarity measurement. A new model based on attention mechanism and weight fusion strategy is proposed in this paper. Firstly, the image is passed through the conv4 network with channel attention mechanism and space attention mechanism to obtain the feature map of the image. On this basis, the fusion strategy is used to extract class-level feature representations according to the difference in contributions of different samples to class-level feature representations. Finally, the similarity scores of query set samples are calculated through the network to predict the classification. Experimental results on the miniImageNet dataset and the omniglot dataset demonstrate the effectiveness of the proposed method.

## Introduction

In recent years, the development of deep learning has been in full swing [1]. It has become a research hotspot in the field of artificial intelligence and has been widely used in computer vision [2, 3], natural language processing [4, 5], video analytics [6, 7], and cyber security [8, 9]. Deep learning is rapidly growing due to the support of big data and the improvement of computing power. In reality, collecting a large amount of labeled data is difficult because of data scarcity or data privacy [10]. At the same time, in the case of sparse data, the traditional deep learning algorithm has been unable to achieve sound classification effects and effective generalization. As for human beings, they have efficient learning abilities and can quickly classify the objects in the pictures after being given one or several images. Furthermore, machines are far worse than humans at this. Hence, few-shot learning has come into being and has become a research hotspot with far-reaching significance and good development prospects. Few-shot learning aims to establish a model with a high generalization ability to have a good classification effect in the case of a few samples [11].

Currently, we can divide few-shot image classification algorithms into three categories [12]: methods based on data enhancement, meta-learning methods, and metric-based methods. According to the idea that traditional deep neural networks rely on big data for training. Data enhancement technology is used to expand the number of samples in few-shot learning. Antoniou et al. proposed DAGAN [13], which learned a large invariance space, trained conditional generative adversarial networks based on the source domain, and employed it in the target domain. In addition, Bateni et al. [14] used unlabelled instances to expand the number of samples and combined them with Mahalanobis distance to improve test image classification accuracy. Dual TriNet [15] used an end-to-end ranking network to perform one-shot learning. However, other methods may depend on semantic attributes [16] or word vectors [17]. Both ways rely on additional information to increase the model parameters. In conclusion, methods based on data augmentation can only partially solve the few-shot learning problem. In contrast, meta-learning methods aim to train a meta-learner so that the model can adapt quickly to different classification tasks and has good generalization performance. Finn et al. proposed a model-agnostic meta-learning algorithm [18], namely MAML. The MAML model trained a meta-learner so that it could quickly find suitable initialization parameters in different classification tasks. Any algorithm optimized by stochastic gradient descent could use the MAML model to achieve better generalization performance. After that, Nichol et al. proposed Reptile [19], an improved version of MAML. Although compared to MAML, the Reptile model needed fewer parameters and could find suitable initialization parameters, improving classification results was less apparent. Ravi et al. proposed Meta-LSTM [20], which used LSTM and experience knowledge to train the meta-learning model. Besides, FEAT [21] used four kinds of set-to-set functions, including BiLSTM [22], DeepSets [23], GCN [24], and Transformer [25], to transform the original embedding feature. Proto-MAML [26] combined the complementary advantages of Prototypical Networks and MAML. In conclusion, Meta-learning methods are based on the future and have some novel ideas, but they are now challenging to apply to practice. The metric-based techniques are simple and efficient, and their core idea is mapping the sample features to the embedded space. With the help of induction bias, the distance function can calculate the similarity among image features to achieve classification. Koch et al. proposed a Siamese Network [27], using two network weight-sharing strategies to extract features from training and test samples. They used euclidean distance to measure the similarity between training and test samples for classification. Furthermore, Vinyals et al. proposed a Matching Network [28], which introduced an attention mechanism to calculate the contribution of training samples to the classification results of test samples so as to complete the classification of test samples. Snell et al. proposed a prototypical network [29], which pushed metric-based methods to a new height. It used the sample mean of all support set samples as the class prototype characterization and measured the similarity between the class prototype characterization and the query samples through the cosine distance to realize the classification. This method ignored the difference in support set samples. Thus, Sung et al. proposed a relation network [30]. This method's most significant improvement was using a neural network as a classifier to calculate the distance between support set samples and query set samples for classification. Apart from these, there were other methods. Kaiser et al. used fast nearest-neighbor algorithms [31] to form a lifelong memory module. It could be easily applied to several networks. Xiao Meng et al. [32] utilized the relationship among the input samples to learn the feature representation and emphasized the importance of feature embedding.

The attention mechanism is one of the core techniques of deep learning. The core idea of the attention mechanism is to accurately distinguish the importance of different regions in the image features so that the model focuses on the areas that influence the classification results and weakens the attention to the outside areas. Sitaula et al. used a novel attention-based deep learning model for diagnosing COVID-19 disease [33]. Because the model was concerned about spatial relationship of CXR images, experiment results proved that the method was suitable for CXR image classification. SE-Net [34] was proposed by Hu Jie et al. in the same year, they won the ImageNet classification contest using SE-Net. Therefore, the network can get a good classification effect by introducing it into a convolutional neural network. Then, other researchers successively proposed CBAM [35], SK-Net, DA-Net and Pyramid feature attention network [36], and ResNet [37]. These attention mechanisms can greatly improve classification accuracy. The plug-and-play feature of the attention model is convenient for model design and can significantly improve the training accuracy of the model. The training samples of few-shot learning are very few. If we use the attention mechanism to focus on the critical areas of images quickly, the classification effect can be as good as possible in the case of limited training samples.

Based on the above analysis, our contributions are as follows in this paper:

- Because the typical Conv4 network fails to capture the critical area of the sample, we embed the attention module into the Conv4 convolutional network to form a new embedded module to enrich the image feature information.
- This paper proposes a weight fusion module that can clearly distinguish the difference in the contribution degree of each training sample to the test sample classification results under the same task.
- In the classification process, the fixed distance measurement method is simple and direct, and the quality of its feature extraction stage directly affects the final classification effect. Therefore, this paper takes a neural network as a measurement module to improve classification accuracy.

In this paper, the proposed method is used to do experiments on the miniImageNet and omniglot datasets. The results show that the classification accuracy of the proposed method is obviously improved. This proves the effectiveness of the proposed method.

This paper is organized as follows. “Methods” section introduces the definition of few-shot learning and the whole network structure, which consists of the embedding module, the weight fusion module, and the measurement module. “Experiment” section describes experiment datasets and experimental settings. “Results and discussion” section presents experimental results and demonstrates the effectiveness of the proposed modules using ablation experiments. “Conclusions” section summarizes the whole paper and looks into the future.

## Methods

### Situational training mechanism

The partitioning of datasets in few-shot learning is based on task-driven. Each scenario is called a task. During the training phase, samples are usually randomly selected from the training set to form a support set ${\mathcal{D}}_{\textrm{support}}$ and a query set ${\mathcal{D}}_{\textrm{query}}$(${\mathcal{D}}_{\textrm{support}}\cap {\mathcal{D}}_{\textrm{query}}=\varnothing$). *N* categories are randomly selected from the training set, and *K* samples are randomly selected from each category to form the Support Set, namely, $\textrm{Support}\ \textrm{Set}={\left\{\left({x}_i,{y}_i\right)\right\}}_{i=1}^{N\times K}$. From the remaining samples of *N* categories, *K*^′^ samples are randomly selected to form the Query Set, namely, $\textrm{Query}\ \textrm{Set}={\left\{\left({x}_j,{y}_j\right)\right\}}_{j=1}^{N\times {K}^{\prime }}$. Therefore, the number of support set samples is *N* × *K*, and the number of query set samples is *N* × *K*^′^. We call this scenario *N* − way *K* − shot mode.

### Network structure

**Fig. 1:**
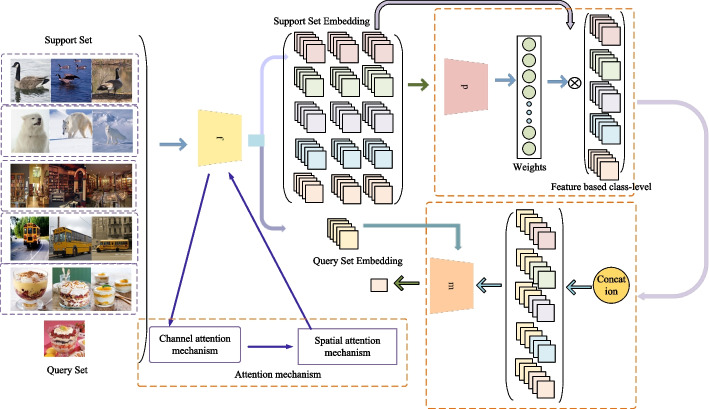
The network structure

The network proposed in this paper consists of three parts: an embedding module, a weight fusion module, and a similarity measurement module. This paper uses Conv4 based on the attention mechanism as the primary network structure. The Conv4 network structure is simple, and the number of parameters is small. In this paper, each intermediate feature is first obtained through the channel attention mechanism. After that, we can get the channel attention feature map and obtain the vital discriminant information of the channel. Then, the attention feature map is obtained through the spatial attention mechanism. The feature representation of each category through the weight fusion module is obtained. This method makes the class-level feature representation more specific and expressive. This paper uses a neural network composed of two convolutional layers and two full connection layers as a classifier. After fusion, the class-level feature characterization and samples of query set are input into the classifier. We can get the final category of samples according to their correlation scores. Figure 1 shows the network structure. This paper’s sections “Embedded module”, “Embedded module”, and “Similarity measurement module” will respectively describe the embedding module f, the weight fusion module p, and the similarity measurement module m.

#### Embedded module

The attention mechanism in neural networks is derived from the human visual mechanism. Given a picture, humans tend to quickly and accurately capture the most valuable areas of the image. Under the problem of image classification in computer vision, researchers often introduce the attention mechanism into the neural network, aiming at making the machine focus on the more discriminative and representative parts of the image so that the model can achieve good classification performance. Embedd module is an important part of a model. Sitaula et al. proposed a novel concept—hybrid deep features [38]. They mixed object-based features and scene-based feature and realized promising classification accuracy. The authors also used content features and context features [39] for scene image representation. After that, Sutaula et al. used VGG-16 architectures pre-trained on datasets for the extraction of foreground, background, and hybrid information [40]. They got the state-of-the-art classification performance.

In this paper, the attention mechanism is integrated into Conv4 to obtain more abundant image feature information. When the number of sample data is minimal, it is crucial to ignore the image's background information and focus on the region of interest of the sample to improve the classification performance. In addition, the attention mechanism is divided into channel attention mechanism, spatial attention mechanism, and mixed attention mechanism. This paper integrates the channel and spatial attention mechanisms into Conv4 to obtain more abundant image feature information. According to CBAM, we should extract the channel and spatial features in succession. In this paper, we first use the channel attention mechanism and then finally extract the embedding features through the spatial attention mechanism.

**Fig. 2:**
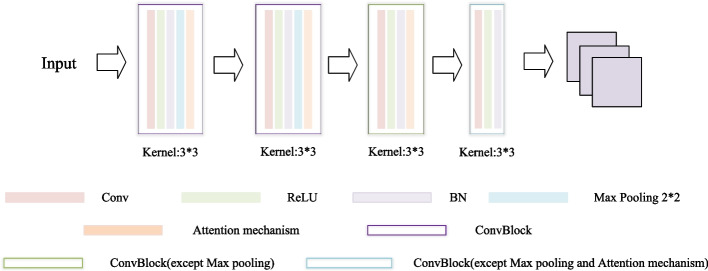
The structure of the embedded module

The Conv4 consists of four convolution blocks. Each block contains a convolution layer, a batch normalization layer, and a ReLU nonlinear layer. The convolution layers are composed of 3 × 3 convolution kernels with 64 channels. The first two convolution blocks respectively add a 2 × 2 max pooling layer. This paper adds the proposed attention mechanism to the first three convolution block. Figure 2 shows the model diagram of the embedded module.

A. Channel attention mechanism

This paper uses SE-Net as the model’s channel attention mechanism. The SE-Net block [34] is the critical structure and core part of SE-Net. It means Squeeze and Excitation. SE-Net model mainly consists of a compression layer, activation layer, and weight layer. We suppose that the middle feature graph is *U*, and the dimension of *U* is *H* × *W* × *C*. Firstly, the feature graph U is compressed through the global average pooling layer to obtain a channel descriptor of 1 × 1 × *C*, which is shown in formula 1.

1 $${z}_c=\frac{1}{H\times W}\sum_{i=1}^H\sum_{j=1}^W{U}_c\left(i,j\right)$$

According to the information in the compression operation, the activation operation is carried out through two full connection layers with the Sigmoid activation function and ReLU activation function, respectively. The purpose is to activate the critical information in the image channel and ignore the invalid data. Formula 2 shows the activation operation.

2 $${S}_c=\sigma \left({W}_2\delta \left({W}_1z\right)\right)$$

Finally, *U*’s channel attention feature map is obtained by multiplying the intermediate input feature map *U* and the output results of the second full connection layer. Equation 3 shows the process.

3 $${X}_c={S}_c\times {U}_c$$

Therefore, the channel attention feature map with more abundant information is obtained through SE-Net.

B. Spatial attention mechanism

The channel attention mechanism only focuses on information between channels, and the feature representation needs to be more comprehensive. Spatial attention mechanisms can help images find the weight of spatial dimensions. On the premise of introducing the channel attention mechanism and combining the spatial attention mechanism, multi-dimensional information fusion is carried out on the feature graph to extract more comprehensive features. In addition, due to many parameters in the two full connection layers of the SE-Net model described in “Channel attention mechanism” section, the spatial attention model proposed in this paper consists of a 1 × 1 convolution kernel and a sigmoid function. This model balances the network parameters and maximizes the performance of the embedded module. The 1 × 1 convolution kernel with one channel compresses the channel dimension of the image, and then the spatial attention weight *s*_*s*_ is obtained.

The calculation process of spatial attention weight *S*_*s*_ is shown in equation 4.

4 $${S}_s=\sigma \left( Conv\left({X}_c\right)\right)$$

The convolution operation reduces the dimension of C channels in the input feature graph. The calculation process is shown in Eq. 5.

5 $$F={X}_c\bigotimes {S}_s$$

**Fig. 3:**
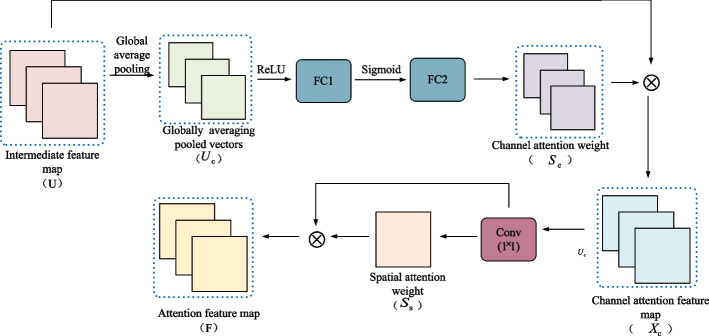
The structure of the attention mechanism

As seen above, the spatial attention mechanism model proposed in this paper has a simple structure. It does not introduce additional parameters, and the embedded module parameters are balanced. Combined with the SE-Net model mentioned in “Channel attention mechanism” section, valuable multidimensional feature information of the original input image can be extracted, which plays an important role in subsequent class-level feature characterization and classification. Figure 3 shows the structure of the attention mechanism.

#### Weight fusion module

**Fig. 4:**
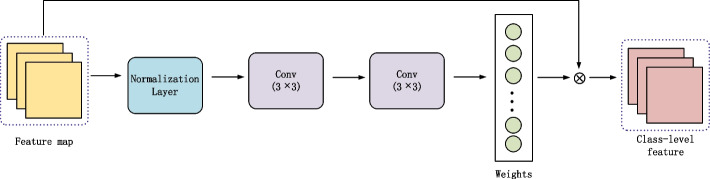
The structure of the weight fusion module

Ideally, the samples of the same class remain clustered in the embedded space. In reality, some deviated instances will inevitably interfere. In the prototypical network, a single support set sample mean is used as the class-level feature representation, and the positive and disturbing samples are treated equally. However, different support set samples have different perspectives. We should treat them differently. This paper proposes a weight fusion module to reduce the bias, weak the contribution of the interference samples to the class-level feature representation, and give more weight to the positive samples. Figure 4 shows the weight fusion module.

The module’s input is the embedded module's output. The structure consists of a regularization layer and two convolution layers. Equation 6 shows the process of the feature fusion module.

6 $$Y={p}_c\left({p}_c\left({p}_n(F)\right)\right)$$

The normalization process of features is shown in Equation 7.

7 $$\left\{\begin{array}{c}\mu =\frac{1}{m}\sum_{i=1}^m{X}_i\\ {}\genfrac{}{}{0pt}{}{\sigma^2=\frac{1}{m}\sum_{i=1}^m{\left({X}_i-\mu \right)}^2}{\hat{X_i}=\frac{X_i-\mu }{\sqrt{\sigma^2-\varepsilon }}}\\ {}{X}_i^{\prime }=\alpha \hat{X_i}+\beta \end{array}\right.$$

Where *X*_*i*_ is the initialization feature, *α*, *β* is the learnable parameter, *μ* is the feature mean, *σ* is the standard deviation, *m* is the number of homogeneous support set samples, and $\hat{X_i}$is the regularization feature. *ε* is 10^−5^.

The weight of each sample in the class is obtained through 3×3 convolution kernels with 64 channels. The weight and the input sample features are weighted and summarized to obtain the class-level feature characterization.

#### Similarity measurement module

In the existing few-shot learning, many typical networks use fixed distance measurement to measure the distance between the query set and the class-level feature representation. Commonly used distance measurement methods are cosine similarity [28] and Euclidean distance [29]. These distance functions cannot be flexibly applied, which affects the model’s performance to some extent. Therefore, this paper uses a neural network for distance measurement. The class-level feature representation and the sample features of the query set are deeply cascaded and input into the measurement module to generate a 0-1 similarity score.

**Fig. 5:**
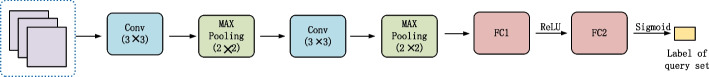
The structure of the similarity measurement module

The structure consists of two convolution layers, two max-pooling layers, and two full connection layers. The convolution layers are composed of 3×3 convolution kernels with 64 channels. The first full connection layer uses the relu activation function, and the second uses the sigmoid activation function. Figure 5 shows the model of the similarity measurement module in this paper.

The algorithm process is as follows:

**Figure:**
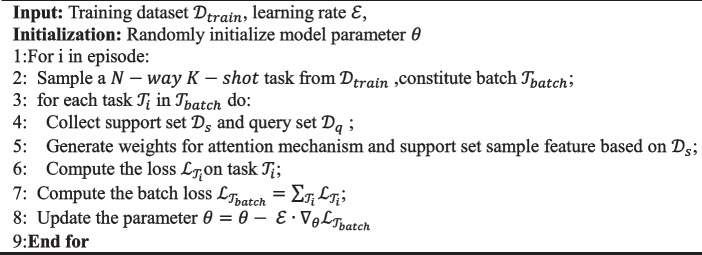
**Algorithm 1 **Training algorithm of the proposed method for N-way K-shot tasks

## Experiment

### Dataset

This experiment uses two reference datasets in few-shot learning: the omniglot dataset [41] and the miniImageNet dataset [28]. The omniglot dataset contains a total of 1623 different handwritten characters from 50 different letters. Each character is drawn online by 20 different people on Amazon’s Mechanical Turk. We rotate the dataset 90°, 180°, and 270° to expand the dataset, and we adjust the input image to 28 × 28. The miniImageNet dataset is divided from the ImageNet dataset. The miniImageNet dataset has 60,000 color images, 100 images in each category. There are 100 categories in total. Each picture size is 84 × 84. This experiment follows the usual few-shot dataset setup, using 1200 classes in the omniglot dataset for training and 423 classes for testing. We use 64 classes for training, 16 classes for validation, and 20 classes for testing in the miniImageNet dataset.

### Configuration of experiment

This paper uses the open framework Pytorch for experiments on Windows 10 operating system and completes a total of 20,000 rounds of training. The initial learning rate is set as 10^−3^ during the training, and the learning rate is halved after every 5000 rounds. In this paper, we use the Adam algorithm as the optimizer. We conduct more than 600 tests with 95% confidence intervals to obtain classification results. The momentum is 0.5, and the weight attenuation coefficient is 0.0005. We use the same settings across all datasets. We also use the cross-entropy loss between the predicted label and its ground truth as a criterion to update parameters. The cross-entropy loss is as follows:

8 $${\mathcal{L}}_{{\mathcal{T}}_i}=H\left({y}_{pre},{y}_{tru}\right)$$

This paper adopts the same few-shot learning experimental settings for training and testing. For the miniImageNet dataset, two training modes are 5-way 1-shot and 5-way 5-shot. In the 5-way 1-shot experiment, each class has one sample in the query set, so there are 5 × 1 + 5 × 1 = 10 samples in a training task. In the 5-way 5-shot experiment, each class contains five samples in the query set, so there are 5 × 5 + 5 × 5 = 50 samples in a training task. For the omniglot dataset, two training modes are 20-way 1-shot and 20-way 5-shot. In the 20-way 1-shot experiment, each class has one sample in the query set, so there are 20 × 1 + 20 × 1 = 40 samples in a training task. In the 20-way 5-shot experiment, each class contains five samples in the query set, so there are 20 × 5 + 20 × 5 = 200 samples in a training task. The experimental settings are shown in Tables 1 and 2.

**Table 1:** MiniImageNet experiment settings

MiniImageNet			
N-way K-shot settings	Support set	Query set	Sum
5-way 1-shot	5×1	5×1	10
5-way 5-shot	5×5	5×5	50

**Table 2:** Omniglot experiment settings

Omniglot			
N-way K-shot settings	Support set	Query set	Sum
20-way 1-shot	20×1	20×1	40
20-way 5-shot	20×5	20×1	200

## Results and discussion

### Experimental results

We use the matching network [28] as the baseline. To verify the effectiveness of the proposed method, the backbone networks of the comparison methods listed in the table are all Conv4. The experimental results are compared with the classification accuracy of MAML [18], matching network [28], prototypical network [29], and relation network [30] in the experiment. We compare this primarily with metric-based approaches. Tables 3 and 4 show the classification accuracy in the miniImageNet and omniglot datasets in this paper.

**Table 3:** Classification accuracy of few-shot image on the miniImageNet dataset (%)

Module	Backbone	5-way classification accuracy	
		1-shot	5-shot
MAML [18]	Conv4	48.70 ±1 .84	63.11 ± 0.92
MATCHING NET [28]	Conv4	43.56 ± 0.84	53.11 ± 0.73
PROTOTYPICAL NET [29]	Conv4	49.42 ± 0.78	68.20 ± 0.66
RELATION NET [30]	Conv4	50.44 ± 0.82	65.32 ± 0.70
GNN [42]	Conv4	50.3	66.4
Meta-Learning LSTM [20]	Conv4	43.44 ± 0.77	60.60 ± 0.71
BOIL [43]	Conv4	49.61 ± 0.16	66.45 ± 0.37
**Our method**	Conv4	**54.16** ***±*** **0.66**	**68.28*±*** **0.71**

**Table 4:** Classification accuracy of few-shot image on omniglot dataset (%)

Module	Backbone	20-way classification accuracy	
		1-shot	5-shot
MA M[18]	Conv4	95.8±0.3	98.9±0.2
MATCHING NE T[28]	Conv4	93.5	98.7
PROTOTYPICAL NE T[29]	Conv4	96.0	98.9
RELATION NE T[30]	Conv4	97.6 ± 0.2	99.1 ± 0.1
**Our method**	Conv4	**97.8**	**99.2**

The above two tables show that the method adopted in this paper has good classification performance on the miniImageNet dataset and omniglot dataset. In the 5-way 1-shot setting of the miniImageNet dataset, the proposed method improves by about 10.6% over the matching network. That is at least about 4% better than the other methods. In the 5-way 5-shot setup of the miniImageNet dataset, the proposed method improves by about 15.1% over the matching network. That is at least about 3% better than the other methods. In the 20-way 1-shot and 20-way 5-shot tasks on the omniglot dataset, the present method improves by 0.2% and 0.1%, respectively. MAML and matching network use fine-tuning strategies, but their results are unsatisfactory. This paper has no experimental fine-tuning procedure, but the classification results are promising. The experimental results show the validity of the model proposed in this paper. Our Conv4 integrates the attention mechanism, inhibits the interference information. We also uses the weight fusion strategy to extract the class features. Therefore, the article can obtain better classification performance. The omniglot dataset is simple, so the accuracy improvement is slight. The miniImageNet dataset is more prosperous than the omniglot dataset, so the accuracy is improved significantly.

### Ablation experiment

#### Experimental analysis of embedded module

This section analyzes the effectiveness of the embedded module combined with the attention mechanism. We compare it with the traditional Conv4 network. Table 5 shows that the embedded module used in this method has improved the classification accuracy well. Because in few-shot learning problems, the number of samples is small, and some images are greatly disturbed by background, the attention mechanism can focus on the vital discriminant regions in samples and quickly capture the most representative sample features. The embedded module combined with the attention mechanism can better extract the sample features that contribute to the accuracy of the few-shot image classification task.

**Table 5:** Comparison of the embedded module in miniImageNet dataset (%)

Image feature	5-way classification accuracy	
	1-shot	5-shot
Ours method (Conv4)	53.76	66.23
Ours method (Conv4+Attention mechanism)	**55.08**	**67.15**

#### Experimental analysis of weight fusion module

This section analyzes the effectiveness of the weight fusion module. We compare it with the mean value of sample features commonly obtained in few-shot learning. It can be seen from Table 6 that the weight fusion module used in this method can sufficiently express the contribution of different samples to the characterization of class-level features. Thus, this method improves the classification accuracy of few-shot images to a certain extent. Because of the noticeable intra-class differences of samples, the contribution degree of some instances that deviate from the class-level feature characterization is not equivalent to that of the adjacent pieces. The class-level feature characterization module proposed by this paper can distinguish the contribution of different samples to the class-level feature characterization and obtain the class-level feature characterization more suitable for a specific task.

**Table 6:** Comparison of weight fusion module in miniImageNet dataset (%)

Class-level feature characteristic	5-way 5-shot classification accuracy
Our method (mean)	64.67
Our method (weight fusion)	**65.92**

#### Experimental analysis of similarity measurement module

This section analyzes the effectiveness of the similarity measurement module and compares it with the fixed distance measure methods. Table 7 shows that this method provides a better classifier, and the neural network as a classifier has a higher classification accuracy. Because fixed distance measurement methods lack flexibility to classify test samples, this method relies heavily on the feature information extracted by the embedding modules. The proposed method uses a neural network as a classifier, which can dynamically classify different samples and preferably learn the similarity between features.

**Table 7:** Comparison of similarity measurement module in miniImageNet dataset (%)

Similarity measurement module	5-way classification accuracy	
	1-shot	5-shot
Our method (cosine distance)	46.10	60.37
Our method (euclidean distance)	48.04	63.48
Our method (neural network)	**54.16**	**68.28**

## Conclusions

This paper uses a new embedded module with the attention mechanism, which combines the channel and spatial attention mechanisms. The model pays attention to the image's region of interest, learns more detailed sample features, enriches the image feature information extracted by the embedded module, and improves the efficiency of feature extraction. According to the difference in contributions of different samples to class-level feature characterization, we set a weight fusion module to obtain more expressive and robust class-level feature characterization. It effectively reduces the impact on samples with less contribution to classification results and improves the induction ability of the model to different instances. Finally, the classifier constructed by the neural network classifies the sample of the query set so that the embedded module and the weight fusion module carry out end-to-end training. Through the above analysis, the method in this paper solves some shortcomings of the existing model, gets a good classification effect on the miniImageNet and omniglot dataset, and plays an excellent performance. We can use the methods in this paper to guide future work on few-shot learning. In the future, we will further explore the influence of other attention mechanisms on feature extraction and verify them on more datasets to make the model better perform generalization.

## Data Availability

Omniglot can be downloaded at omniglot/python at master brendenlake/omniglot (github.com). MiniImageNet dataset can be downloaded at yaoyao-liu/mini-imagenet-tools: Tools for generating mini-ImageNet dataset and processing batches (github.com).

## Abbreviations

DAGAN: Data augmentation generative adversarial networks
MAML: Model agnostic meta learning
LSTM: Long short-term memory network
FEAT: Few-shot embedding adaptation with transformer
GCN: Graph convolutional network
CXR: Chest X-rays
SE-Net: Squeeze-and-excitation networks
CBAM: Convolutional block attention module
SK-Net: Selective kernel networks
DA-Net: Dual attention network
